# Effects of Thiamin Restriction on Exercise-Associated Glycogen Metabolism and AMPK Activation Level in Skeletal Muscle

**DOI:** 10.3390/nu14030710

**Published:** 2022-02-08

**Authors:** Akiko Sato, Shinji Sato, Go Omori, Keiichi Koshinaka

**Affiliations:** 1 Department of Health and Sports, Faculty of Health Science, Niigata University of Health and Welfare, 1398 Shimami-cho, Kita-ku, Niigata 950-3198, Japan; omori@nuhw.ac.jp (G.O.); kosinaka@nuhw.ac.jp (K.K.); 2 Faculty of Applied Life Sciences, Niigata University of Pharmacy and Applied Life Sciences, 265-1 Higashijima, Akiha-ku, Niigata 956-8603, Japan; sato@nupals.ac.jp

**Keywords:** thiamin, glycogen, exercise, mitochondrial content, AMP-activated protein kinase (AMPK)

## Abstract

This study aimed to investigate the direct influence of a decrease in the cellular thiamin level, before the onset of anorexia (one of the symptoms of thiamin deficiency) on glycogen metabolism and the AMP-activated protein kinase (AMPK) activation levels in skeletal muscle at rest and in response to exercise. Male Wistar rats were classified as the control diet (CON) group or the thiamin-deficient diet (TD) group and consumed the assigned diets for 1 week. Skeletal muscles were taken from the rats at rest, those that underwent low-intensity swimming (LIS), or high-intensity intermittent swimming (HIS) conducted immediately before dissection. There were no significant differences in food intake, locomotive activity, or body weight between groups, but thiamin pyrophosphate in the skeletal muscles of the TD group was significantly lower than that of the CON group. Muscle glycogen and lactate levels in the blood and muscle were equivalent between groups at rest and in response to exercise. The mitochondrial content was equal between groups, and AMPK in the skeletal muscles of TD rats was normally activated by LIS and HIS. In conclusion, with a lowered cellular thiamin level, the exercise-associated glycogen metabolism and AMPK activation level in skeletal muscle were normally regulated.

## 1. Introduction

Thiamin is a water-soluble vitamin that functions as a cofactor for pyruvate dehydrogenase (PDH), one of three enzymes the composing pyruvate dehydrogenase complex (PDC) which is a rate-limiting enzyme in carbohydrate metabolism by catalyzing acetyl-CoA production from pyruvate acid. It is well-known that prolonged lower thiamin intake causes symptoms such as anorexia; weight loss; abnormal behavior; and serious diseases, including beriberi and Wernicke-Korsakoff syndrome. Since the amount of thiamin in human body is small and its half-life is short (9–18 days), a decrease in cellular thiamin can occur when the external thiamin intake is limited [1]. On a regular diet, there is little possibility of inducing serious thiamin deficiency-associated diseases because they require time to develop. However, given the thiamin imbalance between supply and demand theoretically precedes the incidence of a serious disorder, and the metabolic disturbance associated with thiamin imbalance possibly appears without noticeable symptoms. This “creeping change” would affect the carbohydrate metabolism especially in athletes and physically active subjects, who are engaging in vigorous training programs and in specific nutritional strategies. It is reported that physical activity may reduce the body’s thiamin retention in rodents and athletes [2,3]. Additionally, in the strategic diet period, i.e., glycogen loading, athletes consume considerably refined carbohydrates. These circumstances may lead to a negative thiamin imbalance in the cell, absolutely and relatively, and then could affect the exercise performance.

Several studies have examined the effect of additional thiamin administration to subjects/animals in normal thiamin state with exercise-induced fatigue and related exercise performance [4,5,6,7,8]. However, despite the nutritional importance of thiamin in carbohydrate metabolism, little is known about the metabolic impact of a negative thiamin imbalance on the exercise performance. Of the few studies related to this issue, a series of studies by the same group of researchers investigated the effect of multivitamin restriction including thiamin on the physical performance of healthy male volunteers. The 8-week experiment resulted in a significant reduction in the maximal oxygen consumption, anaerobic threshold, and onset of blood lactate accumulation in the vitamin-restricted group [9,10,11]. These results indicated that a lower thiamin level in cells was one of the detrimental factors for exercise performance, although the relative contribution of thiamin restriction alone was not clear in those studies.

In addition to the above human study, a recent rodent study by Hernandez-Vazquez et al. [12] examined the effect of thiamin restriction on the glycogen level. It is well known that muscle glycogen is a main energy source during exercise by continuously providing glucose-6-phosphate in the glycogenolytic pathway and in the subsequent aerobic pathway in mitochondria through PDC catalysis. Therefore, both lower muscle glycogen before exercise (at rest) and a higher rate of muscle glycogen depletion during exercise are regarded as potential causes of muscle fatigue [13]. In the above rodent study, the authors showed that thiamin restriction for 3 weeks resulted in a lower liver glycogen level than that found in the control group at rest [12]. Although muscle glycogen was not measured in the study, it is possible that a thiamin restriction-induced decrease in glycogen might also be observed in muscle glycogen at rest, which could be a negative factor for the exercise performance. Hernandez-Vazquez et al. also provided evidence showing that the basal level of AMP-activated protein kinase (AMPK) in thiamin-restricted liver was increased [12]. It notes in the other study that the activation was also observed in thiamin-restricted skeletal muscle at rest [14]. Given that glycogen has an allosteric inhibitory effect on AMPK [15], this result could be considered as supporting evidence for the expected reduction in glycogen content in thiamin-restricted muscle at rest. In addition, AMPK is activated by exercise and functions in the facilitation of glucose uptake and fatty acid oxidation during exercise. If thiamin restriction-induced AMPK activation is also observed during exercise, this phenomenon could influence the rate of muscle glycogen depletion during exercise. However, to the best of our knowledge, there are no studies on the effect of thiamin restriction on exercise-induced AMPK activation and glycogen metabolism in skeletal muscle during exercise.

Therefore, the purpose of this study was to investigate the effect of a lower thiamin level in skeletal muscle on muscle glycogen metabolism at rest and during exercise by measuring the molecular dynamics. We also aimed to determine the effect of the decrease in cellular thiamin on the basal and exercise-induced AMPK activation levels in the skeletal muscles. Since the glycogen expenditure rate during exercise and the exercise-induced AMPK activation level were dependent on the intensity and duration of the exercise, we used different exercise models, namely low-intensity endurance exercise and high-intensity intermittent exercise. For thiamin restriction, most previous studies used a long-term intervention model, which resulted in anorexia; in other words, a metabolically calorie-restricted condition. However, it is known that the reduced food intake, even one-day food reduction, significantly reduced muscle glycogen [16]. Therefore, to see the effect of cellular thiamin reduction itself on muscle glycogen metabolism, we used a short-term intervention model in avoiding the metabolic response induced by anorexia.

## 2. Materials and Methods

### 2.1. Animals

Male Wistar rats were obtained from CLEA Japan (Tokyo, Japan). Rats were housed individually at a constant room temperature (23 ± 1 °C) in a 12-h-light/12-h-dark cycle (06:00 to 18:00) and were provided standard laboratory chow and water ad libitum.

### 2.2. Experimental Design

Rats were divided into the control diet (CON) group and the thiamin-deficient diet (TD) groups according to their weight on the first day of the experiment, when the standard laboratory chow was changed to the experimental diets. The rats consumed the assigned diets ad libitum for 1 week until the end of the dark cycle (06:00) on the day of dissection. The experimental period was decided based on previous studies [12,17,18,19]. These studies demonstrated that no symptoms of thiamin deficiency such as anorexia, weight loss, and behavior disorders were observed on the 7th day of the intervention with thiamin-deficient diet. During our experimental period, the food intake, locomotor activity, and weight changes were measured. The locomotor activity was measured using a passive infrared sensor system (Supermex; Muromachi Kikai, Tokyo, Japan), and the activity during the daytime (6:00 to 18:00) and nighttime (18:00 to 6:00) were separately counted. On the day of dissection, after fasting for 3 h (09:00), several rats were dissected, while others conducted the exercise described below, which was then followed by dissection. Dissection was performed under anesthesia with volatile isoflurane in accordance with the manufacturer’s instructions (MK-A110D; Muromachi Kikai, Tokyo, Japan). Blood was taken from the tail vein before anesthesia, and the blood levels of glucose and lactate were immediately measured with the hand-held analyzer described below. For determining the plasma concentration of insulin, blood was also obtained from the abdominal aorta during dissection and collected into heparin-containing tubes. Muscles were taken and incubated or clamp-frozen in liquid nitrogen for biochemical analysis.

### 2.3. Experimental Diets

The diet of the CON group was composed of 24.3 g of casein, 5.0 g of sucrose, 52.8 g of α-corn starch, 5.0 g of corn oil, 2.2 g of vitamin mix (AIN76), 5.1 g of mineral mix (AIN76), 0.4 g of dl-methionine, 5.0 g of cellulose, and 0.2 g of chorine bitartrate per 100 g. The diet of the TD group was the same as that of the CON group, except that the vitamin mix (AIN76) was replaced with a vitamin mix that was deficient in thiamin. All materials were obtained from the Oriental Yeast (Tokyo, Japan). The diet was isocaloric (3.9 kcal/g). Proteins, fats, and carbohydrates provided 27%, 12%, and 61% of the total energy, respectively.

### 2.4. Exercise

Low-intensity swimming (LIS) corresponded to a 1-h period of swimming with a weight equal to 2% of their body weight [20]. High-intensity intermittent swimming (HIS) consisted of eight 20-s bouts of swimming loaded with a weight equal to 18% of their body weight, with 40 s of rest between the bouts [16,21]. In both patterns of exercise, the rats were accustomed to the swimming exercise for 10 min per day without weights for 2 days before dissection. Water temperature during all swimming exercises was maintained at 35 ± 1 °C. The swimming area and depth of the barrel were controlled to impose equal tasks on all rats [16].

### 2.5. Glucose Transport

#### 2.5.1. Muscle Preparation

The epitrochlearis muscles were incubated with shaking for 20 min at 30 °C in 4 mL of oxygenated Krebs-Henseleit buffer (KHB) containing 40 mM of mannitol and 0.1% radioimmunoassay-grade bovine serum albumin (BSA). Flasks were gassed continuously with 95% O_2_ and 5% CO_2_ during incubation with or without 10 mU/mL of purified human insulin to observe the effect of maximum insulin stimulation as the insulin responsiveness. 

To study the effect of hypoxia, some of the epitrochlearis muscles were incubated for 80 min at 35 °C in hypoxygenated KHB containing 8 mM of glucose, 32 mM of mannitol, and 0.1% BSA under hypoxic conditions (95% N_2_ and 5% CO_2_) [22] before a 15-min incubation at 30 °C in the same oxygenated buffer without insulin, as described above. 

#### 2.5.2. Transport Assay

To measure the rate of muscle glucose uptake, 2-deoxyglucose (2DG) was used according to a previously described method [23]. After a 20- or a 15- min incubation described above, the basal and insulin- or hypoxia-stimulated muscles were further incubated for 20 min at 30 °C in 4 mL of oxygenated KHB containing 8 mM of 2DG, 32 mmol/L of mannitol, and 0.1% BSA with or without insulin at the same concentration as in the initial incubation. The flasks were continuously gassed with 95% O_2_ and 5% CO_2_ during incubation. After incubation, the muscles were blotted dry and clamp-frozen in liquid nitrogen for the fluorometric measurement of 2DG-6-phosphate (2DG6P). 

### 2.6. Biochemical Assay

#### 2.6.1. Muscle Metabolites

Thiamin pyrophosphate (TPP) concentration in the skeletal muscles was determined through a modification of the LC-ESI-MS/MS assay of Roelofsen-de Beer et al. [24], with the stable isotope of thiamin-d3 pyrophosphate (TPP-d3) (Toronto Research Chemical Inc., North York, ON, Canada) as the internal standard. The extensor digitorum longus muscle was weighed in a glass tube with the internal standard solution and homogenized in 0.3 M of perchloric acid (PCA). This mixture was neutralized by KOH and centrifuged at 1000× *g* for 10 min at 4 °C, and the supernatant was injected into the LC-MS/MS system. Mass fragmentography was performed on a Nexera X2 LC-30AD liquid chromatograph, an LCMS-8030 mass spectrometer, and an SIL-30AC auto sampler (all were manufactured by Shimazu Corporation, Kyoto, Japan). The chromatographic column was a Symmetry C18 column (2.1 mm × 100 mm × 3.5 mm) with a column temperature of 40 °C. Isocratic elution using 0.1% formic acid in water as solvent A and 0.1% formic acid in methanol as solvent B (95% A and 5% B) was performed. TPP and TPP-d3 were measured by electrospray ionization in the positive ionization mode with the following selected reaction monitoring mass transitions: m/s 425 > 122 for TPP and m/s 428 > 125 for TPP-d3.

For the measurement of glycogen, lactate, and 2DG6P in skeletal muscle, epitrochlearis muscles were homogenized in 0.3 M of PCA, and the extracts were used to measure glycogen by the amyloglucosidase method [25]. The aliquots were neutralized by KOH and centrifuged at 1000× *g* for 10 min at 4 °C, followed by fluorometric measurements of lactate [26] and 2DG6P [26].

#### 2.6.2. Blood Parameters

The blood glucose and lactate levels were measured using Glutest Every (Sanwa Kagaku Kenkyusho, Nagoya, Japan) and Lactate Pro (Arkray, Kyoto, Japan), respectively. The plasma insulin concentrations were measured using enzyme immunoassay kits (Morinaga Institute of Biological Science, Yokohama, Japan). 

#### 2.6.3. Immunoblot Analysis

The epitrochlearis muscles were homogenized in ice-cold buffer containing 50 mM of HEPES (pH 7.4), 150 mM of NaCl, 10% glycerol, 1% Triton X-100, 1.5 mM of MgCl_2_, 1 mM of EDTA, 10 mM of Na_4_P_2_O_7_, 100 mM of NaF, 2 mM of Na_3_VO_4_, 2 mM of PMSF, aprotinin (10 μg/mL), and leupeptin (10 μg/mL) [16]. The homogenates were rotated end-over-end at 4 °C for 60 min, and then centrifuged at 10,000× *g* for 10 min at 4 °C. Aliquots of supernatants were used for the immunoblot analysis. Briefly, the supernatants were electrophoretically separated by SDS-PAGE and transferred to polyvinylidene fluoride membranes. The membranes were incubated overnight at 4 °C with antibodies against the total and phosphorylated AMPKα (Thr172), thiamin transporter protein (THTR) 1, citrate synthase (CS), cytochrome C oxidase (COX) IV, peroxisome proliferators-activated receptor-γ co-activator (PGC) -1α, monocarboxylate transporter (MCT) 1, MCT4, glucose transporter (GLUT) 4, and glyceraldehyde-3-phosphate dehydrogenase (GAPDH). They were then incubated for 90 min with appropriate horseradish peroxidase-conjugated IgG. The obtained values of phosphorylated AMPKα were normalized to the total AMPKα abundance, and other proteins were normalized by GAPDH abundance. Antibodies against total and phosphorylated AMPKα, CS, and COX IV were obtained from Cell Signaling Technology (Beverly, MA, USA), while anti-PGC-1α, MCT1, and MCT4 antibodies were obtained from the Merck (Temecula, CA, USA). Anti-THTR1, GLUT4, and GAPDH antibodies were purchased from Alpha Diagnostic International Inc. (San Antonio, TX, USA), Biogenesis (Poole, UK), and Sigma-Aldrich (St. Louis, MO, USA), respectively. Immunoreactive bands were visualized using an enhanced chemiluminescence reagent (GE Healthcare Japan, Hino, Japan) and quantified using ImageJ software (National Institutes of Health, Bethesda, MD, USA).

### 2.7. Statistics

Values were expressed as the mean ± SE. Differences between the groups were determined using the unpaired *t*-test. SPSS version 22.0 J for Windows (IBM Japan, Tokyo, Japan) was used for all statistical analyses. The null hypothesis was rejected at the 0.05 level of probability.

## 3. Results

### 3.1. Thiamin Deficiency-Associated Symptoms and Muscle Thiamin Content

**Figure 1:**
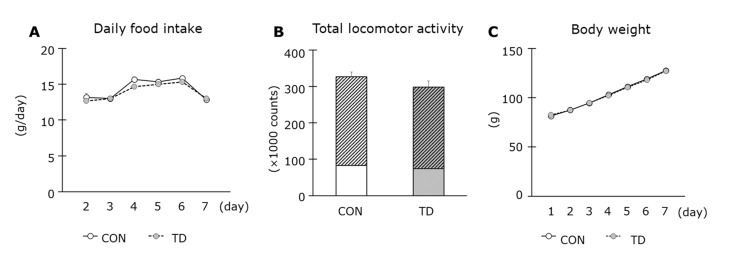
Effects of 1-week thiamin-deficient diet on food intake, locomotor activity, and body weight. Daily food intake (**A**), total locomotor activity (**B**), and body weight (**C**) were measured during the experimental period. In locomotor activity, hatched areas of the bars show values during nighttime (18:00–6:00) and clear areas show that during daytime (6:00–18:00). Values are the means ± SE (*n* = 6–7). CON: control diet, TD: thiamin-deficient diet.

During the experimental period, there was no significant difference in the daily food intake between both groups (Figure 1A). In addition, the total, daytime, and nighttime locomotive activities were equivalent between the CON and the TD groups (Figure 1B). The body weights of the rats in both groups increased at the same rate. At dissection, they became 127.5 ± 2.3 g and 127.2 ± 1.2 g for the CON and the TD groups, respectively, and their weights were not significantly different (Figure 1C). In addition, TD rats showed no visible signs of thiamin deficiency. These results confirmed that TD rats did not present thiamin deficiency-associated symptoms, such as anorexia, abnormal behavior, and weight loss.

**Figure 2:**
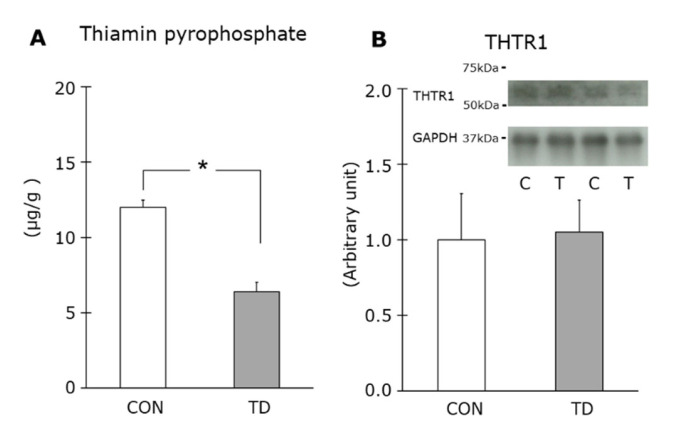
Effects of the 1-week thiamin-deficient diet on the muscle thiamin content and thiamin transporter. Thiamin pyrophosphate content (**A**) and the protein level of thiamin transporter (THTR) 1 (**B**) in skeletal muscle were measured. Representative blots are shown above the figure. Values are the means ± SE (*n* = 4–6). The THTR1 values were normalized by glyceraldehyde-3-phosphate dehydrogenase (GAPDH) abundance. CON, C: control diet, TD, T: thiamin-deficient diet. * *p* < 0.05 vs. TD group.

The TPP content in the skeletal muscles of the TD rats was approximately half of that of the CON rats (*p* < 0.05) (Figure 2A). THTR1 is one of two thiamin specific transporters that are widely distributed in tissues but are most abundant in the skeletal muscles [27,28]. An immunoblot analysis showed that the THTR1 levels in the CON and in the TD groups were equivalent (Figure 2B). The values of GAPDH for the CON and the TD groups were equivalent (1 ± 0.06 vs. 0.94 ± 0.06), confirming that equal amounts of protein were included in the analyzed samples of each group.

### 3.2. Muscle Glycogen and Related Regulations at Rest

**Figure 3:**
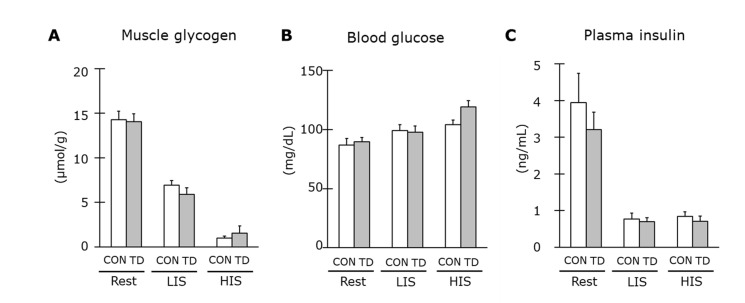
Effects of 1-week thiamin-deficient diet on muscle glycogen and blood parameters at rest and immediately after exercise. Muscle glycogen content (**A**), blood glucose concentration (**B**) and plasma insulin level (**C**) at rest and immediately after low-intensity swimming (LIS) and high- intensity intermittent swimming (HIS) are shown. Values are the means ± SE (*n* = 5–8). CON: control diet, TD: thiamin-deficient diet.

Despite the lowered thiamin level in skeletal muscles, the muscle glycogen content of the CON and the TD groups were equal after a 1-week feeding period (Figure 3A). The glycogen content is controlled by glucose entry into cells with the coordination of substrate delivery and insulin-stimulated glucose transport activity. In this study, the blood glucose concentration (Figure 3B) and plasma insulin level (Figure 3C) were not significantly different between the groups. 

**Figure 4:**
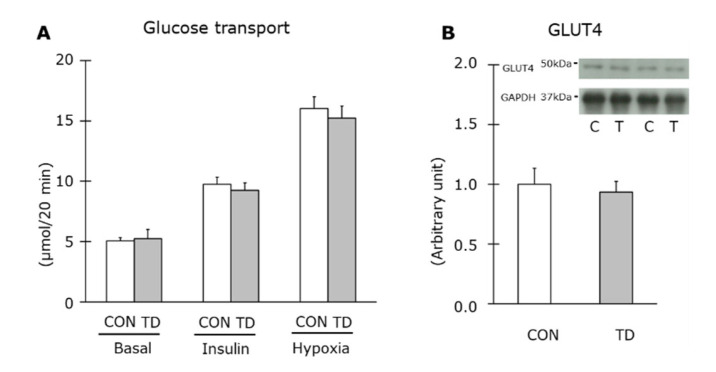
Effects of 1-week thiamin-deficient diet on glucose transport and glucose transporter in skeletal muscle. Glucose transport of skeletal muscle (**A**) was measured under the basal, insulin-, and hypoxia-stimulated conditions. The protein level of glucose transporter (GLUT) 4 (**B**) in skeletal muscle were analyzed by Western blotting, and representative blots are shown above the figure. Values are the means ± SE (*n* = 6–7). The GLUT4 values were normalized by GAPDH abundance. CON: control diet, TD: thiamin-deficient diet.

In addition, glucose transport in the skeletal muscle of both groups was statistically similar under basal and insulin-stimulated conditions. The unchanged glucose transport activity was further confirmed by hypoxic condition, other insulin-independent stimulation (Figure 4A). An immunoblot analysis of GLUT4 showed equal levels of expression in the skeletal muscles of the two groups (Figure 4B). These normality in substrate delivery and glucose transport activity support our results of the glycogen content in thiamin-restricted muscles.

### 3.3. Mitochondria Content and Related Molecule for Mitochondrial Biogenesis

**Figure 5:**
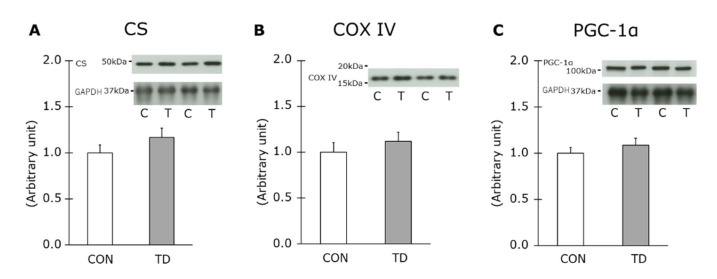
Effects of 1-week thiamin-deficient diet on metabolic molecules related to the mitochondria content in skeletal muscle. The protein levels of citrate synthase (CS) (**A**), cytochrome c oxidase (COX) IV (**B**), and peroxisome proliferator-activated receptor gamma coactivator (PGC)-1α (**C**) were analyzed. CS and COX IV were measured with the same membrane by reprobing. Representative blot are shown above each figure. Values are the means ± SE (*n* = 12). All values were normalized by GAPDH abundance. CON, C: control diet, TD, T: thiamin-deficient diet.

The major indicators of the mitochondrial content are CS and COX IV, which are enzymes in the TCA cycle and in the electron transfer system, respectively. Since AMPK activation stimulates mitochondrial biogenesis [29,30], thiamin restriction could increase the mitochondria content in skeletal muscle if AMPK is activated in the TD group; therefore, it could the affect glycogen metabolism at rest as well as during exercise. The expression of both molecules was not significantly different between the CON and the TD rats (Figure 5A,B). PGC-1α orchestrates a variety of gene expression related to aerobic metabolism, including the enhancement of mitochondrial biogenesis [31]. An immunoblot analysis showed that PGC-1α in the skeletal muscles was equally expressed in both groups (Figure 5C). These results indicated that a reduction in muscle thiamin level did not affect mitochondrial biogenesis.

### 3.4. Muscle Glycogen and Related Regulations Immediately after Exercise

The muscle glycogen content immediately after exercise indicated different characteristics between both exercise models (Figure 3A). However, there was no significant difference between the two groups, which confirmed that glycogen in the TD rats was used in the same way as that in the CON rats during both exercise models. Blood glucose immediately after LIS and HIS (Figure 3B) was maintained at the same level as at rest, while plasma insulin immediately after both exercise models decreased sharply (Figure 3C). However, there were no differences between the two groups for either variable. These results demonstrated that the rate of muscle glycogen depletion was not affected by thiamin restriction regardless of the exercise models.

**Figure 6:**
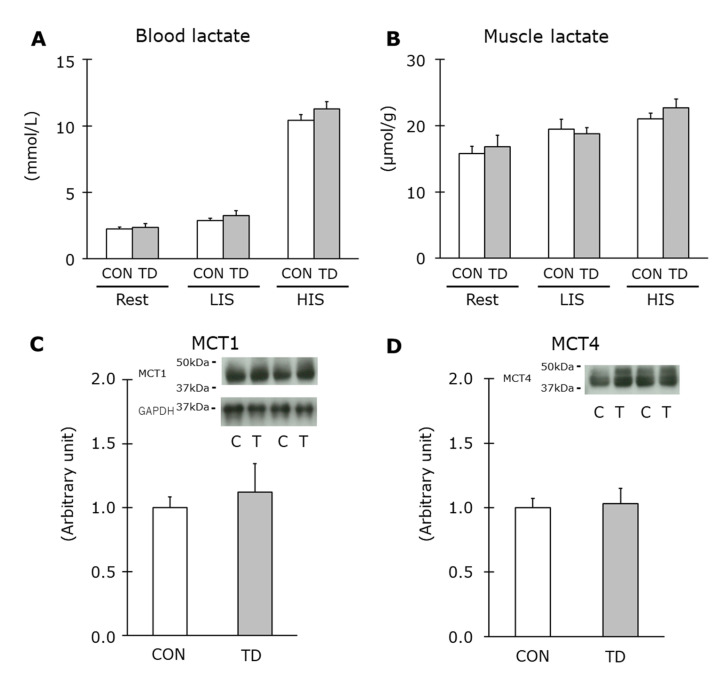
Effects of a 1-week thiamin-deficient diet on lactate and monocarboxylate transporters. Lactate level in the blood (**A**) and in the skeletal muscle (**B**) were measured as well as the protein levels of monocarboxylate transporter (MCT) 1 (**C**), and MCT4 (**D**). MCT1 and MCT4 were measured with the same membrane by reprobing. Representative blots of MCT1 and MCT4 are shown above the figures. Values are the means ± SE (*n* = 5–8). All values were normalized by GAPDH abundance. CON, C: control diet, TD, T: thiamin-deficient diet. LIS: low-intensity swimming, HIS: high-intensity intermittent swimming.

The lactate level is an indicator for the degree of glycolytic flow including glycogen breakdown. Immediately after exercise, the blood lactate level was increased in an exercise intensity-dependent manner compared to the resting condition, but no difference was observed between the CON and the TD groups in any comparisons (Figure 6A,B). MCT1 and MCT4 are transporters that carry monocarboxylates, such as lactate and pyruvate across sarcolemma, and these transporters are responsible for lactate uptake and release, respectively [32,33]. In the immunoblot analysis in this study, both MCT1 and MCT4 proteins of the TD rats were equally expressed compared to those of the CON rats (Figure 6C,D). Taken together, these results of lactate metabolism suggested normal regulation of the glycolytic and glycogenolytic flow during exercise in thiamin-restricted muscles.

### 3.5. AMPK Phosphorylation Levels at Rest and Immediately after Exercise

**Figure 7:**
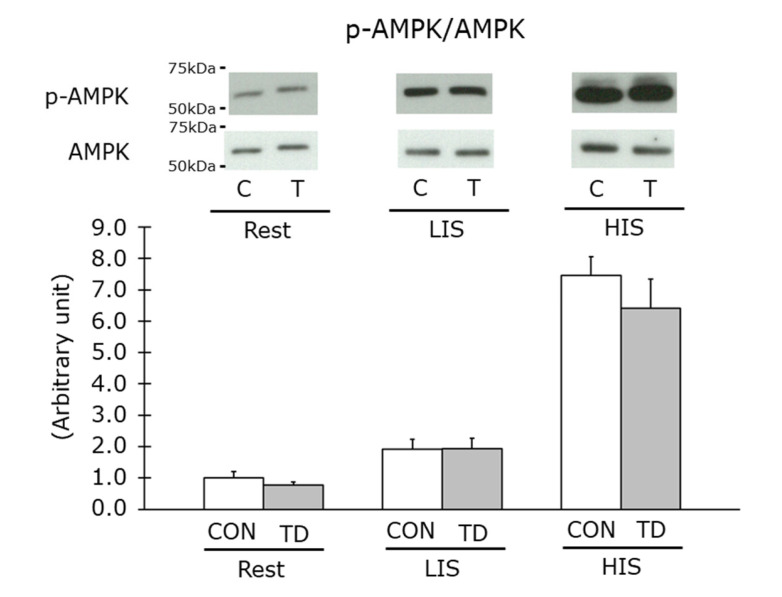
Effects of 1-week thiamin-deficient diet on AMP-activated kinase (AMPK) phosphorylation at rest and immediately after exercise in the skeletal muscle. The values of p-AMPK were normalized by AMPK abundance. Representative blots are shown above the figure. Values are the means ± SE (*n* = 5–7). CON, C: control diet, TD, T: thiamin-deficient diet. LIS: low-intensity swimming, and HIS: high-intensity intermittent swimming.

The AMPK activation levels were measured by immunoblot analysis for the phosphorylated AMPK protein levels. As we expected, exercise amplified the AMPK phosphorylation levels in an exercise intensity-dependent manner. However, in contrast to previous studies [12,14], its phosphorylation level at rest was not increased by thiamin restriction (Figure 7). Additionally, theAMPK phosphorylation levels in response to both exercise models were identical between the CON and the TD groups (Figure 7). 

## 4. Discussion

The reduced food intake, which is a typical symptom in long-lasting thiamin-restricted conditions, is an independent promising factor that affected several impacts on muscle metabolism, including insulin sensitivity, reduction in the muscle glycogen content [16] and stimulation of mitochondrial biogenesis [34,35]. Another symptom, aggressive behavior [36,37], may contribute to these parameters as an exercise-like secondary impact of thiamin deficiency. In contrast to previous studies with long-term intervention models, we chose an experimental model of short-term (1 week) intervention in the present study to avoid the secondary metabolic effects other than the lower levels of cellular thiamin. In our study, thiamin deficiency-associated symptoms did not appear, which was confirmed by the normal food intake, body weight, locomotor activity, and visible appearance. Theoretically nutritional imbalance precedes the incidence of physiological disorder, and this process seems to be on gradual line, presumably with an inflection point to induce symptoms. Therefore, it is not clear and very difficult to judge whether this condition without typical symptoms is in thiamin deficiency or not. However, on the gradual changes, we found that 1-week intervention with thiamin-deficient diet lead to an apparent decrease in the TPP level in the skeletal muscles of TD rats without typical symptoms. In addition, our preliminary study observed the food intake of the TD rats started to decrease after 7-day feeding with thiamin-deficient diet. Thus, 1 week of nutritional intervention was a suitable and the only model to highlight the effect of reduction in cellular thiamin level itself on muscle metabolism.

As observed in a previous study investigating the glycogen content in thiamin-restricted livers [12], we anticipated a lower level of muscle glycogen content in TD rats at the basal level in this study. However, in contrast to this expectation, one of our main results clearly demonstrated for the first time that a 1-week thiamin-deficient diet did not affect the basal glycogen content even when the muscle TPP level was dramatically decreased. In a previous study by Hernandez-Vazquez et al. [12], the authors also found that liver AMPK was activated in thiamin-restricted rats with a reduction of food intake, which potentially was the result of a release from a negative allosteric regulation of AMPK by lowering the glycogen content [15]. In skeletal muscle, a previous study by Liu et al. demonstrated that the AMPK activation level was increased in skeletal muscle of mice fed with thiamin-deficient diet for 4 weeks with anorexic symptom [14]. This result is incompatible with our present result showing that AMPK activation levels in skeletal muscle were identical between the CON and the TD groups when food intake was not different between groups. Taken together, these results suggested that glycogen reduction and AMPK activation at basal level might depend on the anorexic effect of long-term thiamin restriction and that these changes are not inducible by reduced muscle thiamin content itself under non-anorexic condition. 

We further obtained an insight into the additional factors that possibly controlled the basal glycogen levels, namely glucose and insulin delivery to skeletal muscles and insulin-stimulated glucose entry into cells. Regarding these factors, we clearly demonstrated equivalent levels of blood parameters and insulin-stimulated glucose transport with equal levels of GLUT4 expression between the two groups. The mitochondrial content was also examined in this study as a candidate influencer for glycogen metabolism through a glycolytic response to mitochondrial capacity for carbohydrate. However, the level of protein expression of CS, COX IV, and PGC-1α were equal between the CON and the TD rats. These results are supporting our results of the identical basal glycogen level in skeletal muscle between groups.

A similar tendency was observed during exercise, and the muscle glycogen content was equivalent immediately after both LIS and HIS between groups. This suggested that muscle glycogen in both groups was used at the same pace during two different types of exercise, both of which utilize glycogen as the main energy source but their dependency on glycogen for energy production were different. The identical glycogen depletion during the exercise was supported by the equal level of blood and muscle lactate immediately after exercise, with same protein expression levels of MCT1 and MCT4. These results indicated that thiamin-decreased skeletal muscle in this study maintained normal regulation in glycogenolytic and glycolytic metabolism during exercise, at least in a short-term thiamin restriction.

The above discussion on glycogen utilization during exercise was also supported by the results of AMPK, a master switch that regulates substrate utilization and affects glycogen metabolism in skeletal muscle during exercise [38]. Similar to the results at rest, for the first time our immunoblot analysis showed equal phosphorylation of the AMPK level between the CON and the TD rats immediately after both exercise models focusing exercise intensity. These results provided one speculation that continuous training would also induce regular adaptation to exercise with a normal increase in AMPK-associated molecules including the GLUT4, PGC-1α, and MCT proteins [39,40,41,42] as well as mitochondria content in the skeletal muscle of the TD rats.

Thus, the results of this study revealed that even if cellular thiamin levels in skeletal muscles were clearly decreased by a thiamin-deficient diet, regulations of glycogen metabolism and AMPK activation in the skeletal muscle were maintained in response to exercise. A possible explanation for this normal flow of carbohydrate even during exercise might be the very strong impact of exercise stimulation as an activator of PDH. Exercise increases Ca^2+^, ADP, and pyruvate, all of which potently provoke PDH activation in the skeletal muscle independently from thiamin [43], and powerfully stimulates PDH activity [44]. Hence, although PDH activity was not measured in the present study due to our technical limitations, it could be considered that the influence of the lower TPP levels in skeletal muscles on PDH activity might be negated during exercise, regardless of an exercise model.

## 5. Conclusions

In summary, our study demonstrated that a decrease in the muscle thiamin content did not show any influence on muscle glycogen metabolism and AMPK activation levels at rest and in response to exercise, at least before the appearance of thiamin deficiency-associated symptoms. In regard to the optimum thiamin intake and its necessity for physically active population [45,46], it still has been under debate without consideration of any functional and actual metabolic impacts during exercise. Our present results will provide a new insight into dietary thiamin intake and nutritional management for those who engage in physical exercise.

## Data Availability

Not applicable.

## References

[1] Ihara H., Hashizume N. (2011). Nutrition and deficiency: Vitamin B1. The Vitamin Society of Japan eds. General Dictionary of Vitamin.

[2] Shibata K., Fukuwatari T. (2013). The body vitamin B1 levels of rats fed a diet containing the minimum requirement of vitamin B1 is reduced by exercise. J. Nutr. Sci. Vitaminol..

[3] Sato A., Shimoyama Y., Ishikawa T., Murayama N. (2011). Dietary thiamin and riboflavin intake and blood thiamin and riboflavin concentrations in college swimmers undergoing intensive training. Int. J. Sport Nutr. Exerc. Metab..

[4] Suzuki M., Itokawa Y. (1996). Effects of thiamine supplementation on exercise-induced fatigue. Metab. Brain Dis..

[5] Doyle M.R., Webster M.J., Erdmann L.D. (1997). Allithiamine Ingestion Does Not Enhance Isokinetic Parameters of Muscle Performance. Int. J. Sport Nutr..

[6] Doyle M.R., Branz M., Webster M.J., Scheett T.P. (1997). The effect of a thiamin derivative on exercise performance. Graefe’s Arch. Clin. Exp. Ophthalmol..

[7] Nozaki S., Mizuma H., Tanaka M., Jin G., Tahara T., Mizuno K., Yamato M., Okuyama K., Eguchi A., Akimoto K. (2009). Thiamine tetrahydrofurfuryl disulfide improves energy metabolism and physical performance during physical-fatigue loading in rats. Nutr. Res..

[8] Choi S.K., Baek S.H., Choi S.W. (2013). The effects of endurance training and thiamine supplementation on anti-fatigue during exercise. J. Exerc. Nutr. Biochem..

[9] Van der Beek E.J., Van Dokkum W., Schrijver J., Wesstra J.A., Van de Weerd H., Hermus R.J.J. (1984). Effect of marginal vitamin intake on physiological performance in man. Int. J. Sport Med..

[10] Van der Beek E.J., van Dokkum W., Schrijver J., Wedel M., Gaillard A.W., Wesstra A., van de Weerd H., Hermus R.J. (1988). Thiamin, riboflavin, and vitamins B-6 and C: Impact of combined restricted intake on functional performance in man. Am. J. Clin. Nutr..

[11] van der Beek E.J., Van Dokkum W., Wedel M., Schrijver J., van den Berg H. (1994). Thiamin, riboflavin and vitamin B6: Impact of restricted intake on physical performance in man. J. Am. Coll. Nutr..

[12] Hernandez-Vazquez A.D.J., Garcia-Sanchez J.A., Moreno-Arriola E., Salvador-Adriano A., Ortega-Cuellar D., Velazquez-Arellano A. (2016). Thiamine Deprivation Produces a Liver ATP Deficit and Metabolic and Genomic Effects in Mice: Findings Are Parallel to Those of Biotin Deficiency and Have Implications for Energy Disorders. J. Nutr. Nutr..

[13] Ørtenblad N., Westerblad H., Nielsen J. (2013). Muscle glycogen stores and fatigue. J. Physiol..

[14] Liu M., Alimov A., Wang H., Frank J., Katz W., Xu M., Ke Z.-J., Luo J. (2014). Thiamine deficiency induces anorexia by inhibiting hypothalamic AMPK. Neuroscience.

[15] Janzen N.R., Whitfield J., Hoffman N.J. (2018). Interactive Roles for AMPK and Glycogen from Cellular Energy Sensing to Exercise Metabolism. Int. J. Mol. Sci..

[16] Koshinaka K., Kawasaki E., Hokari F., Kawanaka K. (2009). Effect of acute high-intensity intermittent swimming on post-exercise insulin responsiveness in epitrochlearis muscle of fed rats. Metabolism.

[17] Liang C.C. (1975). Metabolic changes in rats during developing thiamin deficiency.(Short Communications). Biochem. J..

[18] Nakagawasai O., Tadano T., Hozumi S., Taniguchi R., Tan-No K., Esashi A., Niijima F., Kisara K. (2001). Characteristics of depressive behavior induced by feeding thiamine-deficient diet in mice. Life Sci..

[19] Gralak M.A., Dębski B., Drywień M. (2019). Thiamine deficiency affects glucose transport and β-oxidation in rats. J. Anim. Physiol. Anim. Nutr..

[20] Koshinaka K., Honda A., Masuda H., Sato A. (2020). Effect of Quercetin Treatment on Mitochondrial Biogenesis and Exercise-Induced AMP-Activated Protein Kinase Activation in Rat Skeletal Muscle. Nutrients.

[21] Kawanaka K., Tabata I., Tanaka A., Higuchi M. (1998). Effects of high-intensity intermittent swimming on glucose transport in rat epitrochlearis muscle. J. Appl. Physiol..

[22] Cartee G., Douen A.G., Ramlal T., Klip A., Holloszy J.O. (1991). Stimulation of glucose transport in skeletal muscle by hypoxia. J. Appl. Physiol..

[23] Hansen P.A., Gulve E.A., Holloszy J.O. (1994). Suitability of 2-deoxyglucose for in vitro measurement of glucose transport activity in skeletal muscle. J. Appl. Physiol..

[24] Roelofsen-de Beer RJ A.C., Van Zelst B.D., Wardle R., Kooij P.G., de Rijke Y.B. (2017). Simultaneous measurement of whole blood vitamin B1 and vitamin B6 using LC-ESI-MS/MS. J. Chromatogr. B Analyt. Technol. Biomed Life Sci..

[25] Passonneau J., Lauderdale V. (1974). A comparison of three methods of glycogen measurement in tissues. Anal. Biochem..

[26] Passonneau J.V., Lowry O.H. (1993). Enzymatic Analysis. A Practical Guide.

[27] Fleming J.C., Tartaglini E., Steinkamp M.P., Schorderet D.F., Cohen N., Neufeld E.J. (1999). The gene mutated in thiamine-responsive anaemia with diabetes and deafness (TRMA) encodes a functional thiamine transporter. Nat. Genet..

[28] Dutta B., Huang W., Molero M., Kekuda R., Leibach F.H., Devoe L.D., Ganapathy V., Prasad P.D. (1999). Cloning of the Human Thiamine Transporter, a Member of the Folate Transporter Family. J. Biol. Chem..

[29] Leick L., Fentz J., Biensø R.S., Knudsen J.G., Jeppesen J., Kiens B., Wojtaszewski J., Pilegaard H. (2010). PGC-1α is required for AICAR-induced expression of GLUT4 and mitochondrial proteins in mouse skeletal muscle. Am. J. Physiol. Metab..

[30] Jäger S., Handschin C., Pierre J.S., Spiegelman B.M. (2007). AMP-activated protein kinase (AMPK) action in skeletal muscle via direct phosphorylation of PGC-1α. Proc. Natl. Acad. Sci. USA.

[31] Austin S., St-Pierre J. (2012). PGC1α and mitochondrial metabolism—Emerging concepts and relevance in ageing and neurodegenerative disorders. J. Cell Sci..

[32] Manning Fox J.E., Meredith D., Halestrap A.P. (2000). Characterisation of human monocarboxylate transporter 4 substantiates its role in lactic acid efflux from skeletal muscle. J. Physiol..

[33] Halestrap A.P., Meredith D. (2004). The SLC16 gene family?from monocarboxylate transporters (MCTs) to aromatic amino acid transporters and beyond. Pflügers Arch.-Eur. J. Physiol..

[34] López-Lluch G., Hunt N., Jones B., Zhu M., Jamieson H., Hilmer S., Cascajo M.V., Allard J., Ingram D.K., Navas P. (2006). Calorie restriction induces mitochondrial biogenesis and bioenergetic efficiency. Proc. Natl. Acad. Sci. USA.

[35] Civitarese A.E., Carling S., Heilbronn L., Hulver M.H., Ukropcova B., Deutsch W.A., Smith S.R., Ravussin E. (2007). Calorie Restriction Increases Muscle Mitochondrial Biogenesis in Healthy Humans. PLoS Med..

[36] Onodera K., Tadano T., Sakai K., Kisara K., Ogura Y. (1978). Muricide induced by thiamine deficiency in the rats. Folia Pharmacol. Jpn..

[37] Vorhees C.V., Barrett R.J., Schenker S. (1979). Thiamin deficiency induced muricide behavior in rats. Physiol. Behav..

[38] Steinberg G.R., Kemp B.E. (2009). AMPK in Health and Disease. Physiol. Rev..

[39] Hardie D.G. (2011). AMP-activated protein kinase—an energy sensor that regulates all aspects of cell function. Genes Dev..

[40] Cantó C., Auwerx J. (2010). AMP-activated protein kinase and its downstream transcriptional pathways. Exp..

[41] Takimoto M., Takeyama M., Hamada T. (2013). Possible involvement of AMPK in acute exercise-induced expression of monocarboxylate transporters MCT1 and MCT4 mRNA in fast-twitch skeletal muscle. Metabolism.

[42] Kitaoka Y., Takahashi Y., Machida M., Takeda K., Takemasa T., Hatta H. (2014). Effect of AMPK activation on monocarboxylate transporter (MCT)1 and MCT4 in denervated muscle. J. Physiol. Sci..

[43] Spriet L.L., Heigenhauser G.J. (2002). Regulation of Pyruvate Dehydrogenase (PDH) Activity in Human Skeletal Muscle During Exercise. Exerc. Sport Sci. Rev..

[44] Howlett R.A., Heigenhauser G.J.F., Hultman E., Hollidge-Horvat M.G., Spriet L.L. (1999). Effects of dichloroacetate infusion on human skeletal muscle metabolism at the onset of exercise. Am. J. Physiol. Metab..

[45] Manore M. (2000). Effect of physical activity on thiamine, riboflavin, and vitamin B-6 requirements. Am. J. Clin. Nutr..

[46] Woolf K., Manore M.M. (2006). B-Vitamins and Exercise: Does Exercise Alter Requirements?. Int. J. Sport Nutr. Exerc. Metab..

